# Interleukin‐4‐loaded hydrogel scaffold regulates macrophages polarization to promote bone mesenchymal stem cells osteogenic differentiation via TGF‐β1/Smad pathway for repair of bone defect

**DOI:** 10.1111/cpr.12907

**Published:** 2020-09-19

**Authors:** Jiankang Zhang, Haitao Shi, Nian Zhang, Liru Hu, Wei Jing, Jian Pan

**Affiliations:** ^1^ State Key Laboratory of Oral Diseases National Clinical Research Center for Oral Diseases Department of Oral and Maxillofacial Surgery West China Hospital of Stomatology Sichuan University Chengdu China

## Abstract

**Objective:**

Tissue engineering is a promising strategy for repair of large bone defect. However, the immune system reactions to biological scaffold are increasingly being recognized as a crucial factor influencing regeneration efficacy. In this study, a bone‐bioactive hydrogel bead loaded with interleukin‐4 (IL‐4) was used to regulate macrophages polarization and accelerate bone regeneration.

**Methods:**

IL‐4‐loaded calcium‐enriched gellan gum (Ca‐GG + IL‐4) hydrogel beads were synthesised. And the effect on cell behaviour was detected. Furthermore, the effect of the Ca‐GG + IL‐4 hydrogel bead on macrophage polarization and the effect of macrophage polarization on bone mesenchymal stem cells (BMSCs) apoptosis and osteogenic differentiation were evaluated in vitro and in vivo.

**Results:**

BMSCs were able to survive in the hydrogel regardless of whether IL‐4 was incorporated. Immunofluorescence staining and qPCR results revealed that Ca‐GG + IL‐4 hydrogel bead could promote M2 macrophage polarization and increase transforming growth factor (TGF)‐β1 expression level, which activates the TGF‐β1/Smad signalling pathway in BMSCs and promotes osteogenic differentiation. Moreover, immunohistochemical analysis demonstrated Ca‐GG + IL‐4 hydrogel bead could promote M2 macrophage polarization and reduce cell apoptosis in vivo. In addition, micro‐CT and immunohistochemical analysis at 12 weeks post‐surgery showed that Ca‐GG + IL‐4 hydrogel bead could achieve superior bone defect repair efficacy in vivo.

**Conclusions:**

The Ca‐GG + IL‐4 hydrogel bead effectively promoted bone defect regeneration via regulating macrophage polarization, reducing cell apoptosis and promoting BMSCs osteogenesis through TGF‐β1/Smad pathway. Therefore, it is a promising strategy for repair of bone defect.

## Graphical abstract

Interleukin‐4‐loaded Ca‐enriched gellan gum hydrogel scaffold could transform macrophages from the proinflammatory M1 phenotype into the anti‐inflammatory M2 phenotype. And reduced BMSCs apoptosis caused by M1 macrophages secreted TNF‐α. And M2 phenotype macrophages accelerate bone regeneration via improving osteogenic differentiation capacity of BMSCs by triggering TGF‐β1/Smad signalling pathway.

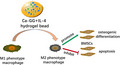

## 1 INTRODUCTION

Large bone defect remains a major clinical problem worldwide, which could be caused by tumour resection, congenital abnormality and trauma. Bone graft therapy is currently the most common clinical treatment.^1^

However, there are several factors that limit the use of this clinical therapy, including bone‐related disorders and limited donor supplies.^2^

Recently, tissue engineering has become a promising approach for large bone defects regeneration.^3^, ^4^ An important issue regarding bone tissue engineering is the construction of biological scaffold with biofunction to modulate cellular behaviour such as proliferation, migration and differentiation.^5^ Because of the capacity of imitating many matrix parameters of natural extracellular matrix (ECM), hydrogel‐based biomaterials have become promising candidates for a large number of tissue engineering applications.^6^ The hydrophilic polymeric networks of hydrogels can retain water without losing their inner structure, and they can also be used as carriers for drug and protein delivery.^7^, ^8^, ^9^ Gellan gum (GG) hydrogel is a biodegradable polysaccharide with high cytocompatibility and has been approved by the United States Food and Drug Administration as a biomaterial.^10^, ^11^, ^12^ Numerous studies on the application of GG hydrogels have been carried out in tissue engineering, including studies on brain tissue engineering and cartilage and intervertebral disc applications.^13^, ^14^ GG hydrogel‐based matrices have great potential for the controlled delivery of stem cells, different drugs and growth factors in situ.^15^ Biomineralization mediated by ionic exchange with the surrounding microenvironment has recently gained substantial attention and has been reported to promote bone mesenchymal stem cells (BMSC) osteogenic differentiation.^16^, ^17^ A previous study showed that Ca‐GG hydrogels exhibited strong mineralization capacity because the incorporated calcium ions facilitate phosphate ion deposition and further mineralization. Furthermore, Ca‐GG hydrogel beads are compatible with a wide range of molecular drug delivery applications.^18^

Macrophages are myeloid precursor cells that derived from bone marrow and are an important part of the body's innate immune system. Macrophages play an important role in inflammation, host defence, tissue repair and metabolism.^19^ Mononuclear macrophages possess the characteristics of diversity and plasticity, and they can differentiate into different phenotypes and play different roles in different microenvironments; this differentiation is called macrophage polarization, and it plays an important role in many physiological and pathological processes.^20^ Studies show that the IRF1/STAT1 signalling pathway, which is activated by interferon (IFN)‐γ and TLRs, can mediate M1 type polarization, while the IRF4/STAT6 signalling pathway, which is activated by IL‐4 and IL‐13, can mediate M2 type polarization.^21^ M1 macrophages are characterized by the high expression of proinflammatory factors and nitrogen oxides, which have a killing effect on microorganisms. M2 macrophages are characterized by the high expression of scavenger molecules and exert highly efficient phagocytic and immunomodulatory functions; M2 macrophages can promote tissue repair.^22^, ^23^, ^24^

The aggregation and differentiation of BMSCs at the site of bone defect and the subsequent formation of bone and cartilage are regulated by multiple factors of the local microenvironment.^25^ The interaction and precise interregulation between macrophages and cells related to bone formation play increasingly important roles in both endochondral and membranous osteogenesis.^26^ Some inflammatory cytokines secreted by M1 macrophages, such as tumour necrosis factor‐α (TNF‐α), could result in BMSCs apoptosis. And some inflammatory cytokines secreted by M2 macrophages could promote the osteogenic differentiation of BMSCs.^27^

In this study, bone‐bioactive Ca‐GG hydrogel beads were successfully synthesized. Interleukin‐4 (IL‐4), one of the cytokines that can regulate the transformation of macrophages from the proinflammatory M1 phenotype into the anti‐inflammatory M2 phenotype,^28^ was incorporated into the hydrogel beads to regulate macrophage polarization and promote bone regeneration. The aqueous and ECM‐like microenvironment in the GG hydrogel bead makes it a suitable IL‐4 delivery vehicle. Then the effects of the Ca‐GG + IL‐4 hydrogel beads on macrophage immunomodulation, BMSCs apoptosis and osteogenic differentiation were evaluated both in vitro and in vivo.

## 2 MATERIALS AND METHODS

### 2.1 Preparation of Ca‐GG + IL‐4 hydrogel beads and conditioned media

**Figure 1:**
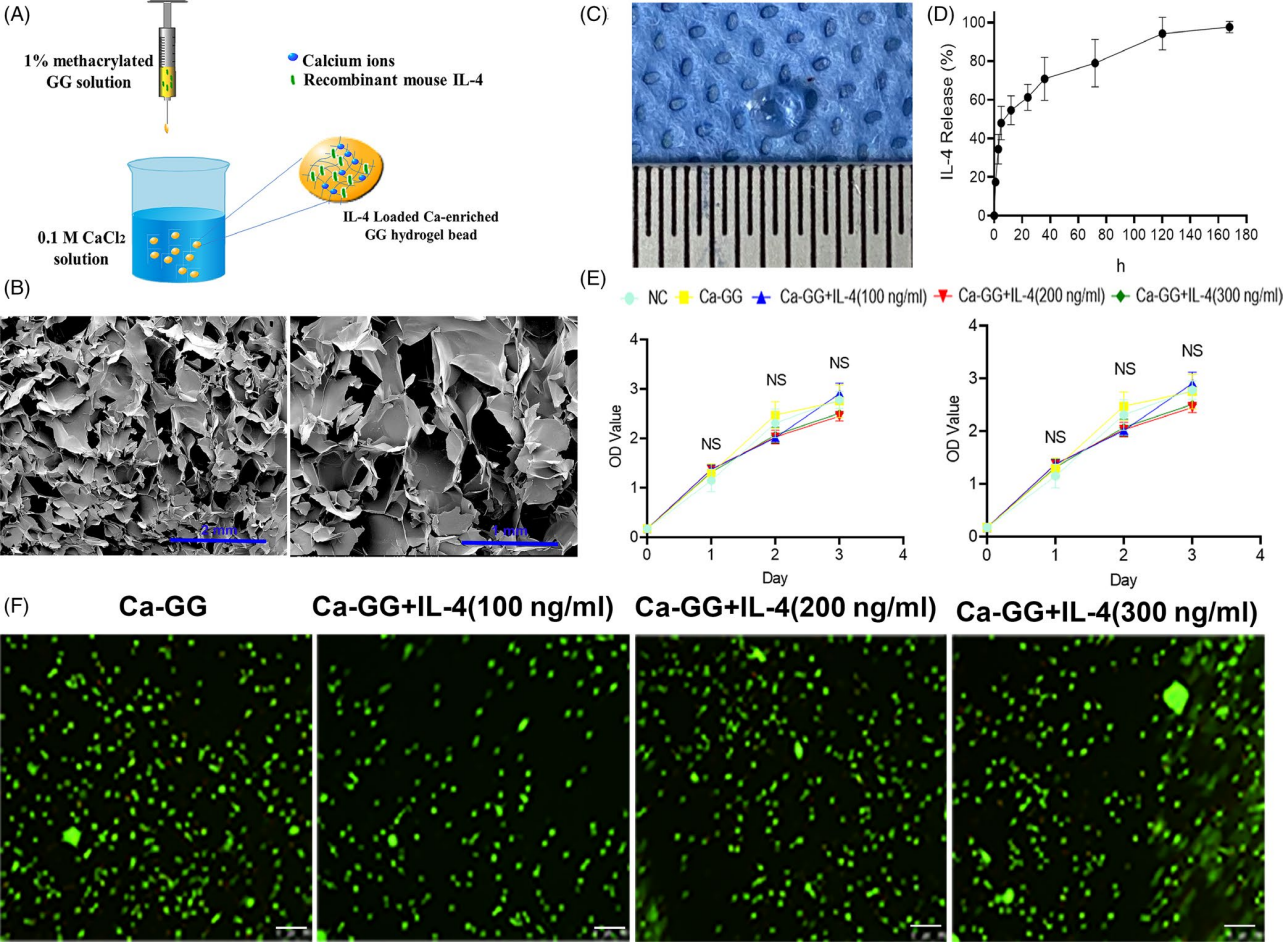
Synthesis process of Ca‐GG hydrogel bead, Viability of cells in Ca‐GG + IL‐4 hydrogel and IL‐4 release assay. A, Schematic diagram of the Ca‐GG hydrogel bead preparation process. B, The micro‐structure of Ca‐GG hydrogel evaluated by SEM. C, The general view of Ca‐GG hydrogel bead. D, In vitro IL‐4 release assay. E, CCK‐8 assay of BMSCs and RAW 264.7 cultured in different conditioned media. F, Fluorescence staining of BMSCs cultured within Ca‐GG + IL‐4 hydrogel over 3 d (scale bars = 250 μm); living cells were labelled with calcein‐AM (green fluorescence), while dead cells were labelled with PI (red fluorescence)

GG powder (0.5 g, Sigma‐Aldrich) and an appropriate volume of glycidyl methacrylate (Sigma‐Aldrich) were added to 50 mL distilled water with continuous stirring at room temperature for 8 hours. Then, cold acetone was used to precipitate the reaction products. Next, the solution was further purified by dialysis and freeze‐dried. Then, dry methacrylated GG was dissolved in distilled water to form a 1% (w/v) GG solution. Then, 100, 200 or 300 ng recombinant mouse IL‐4 was added to 1 mL GG solution, respectively. The 0.1 mol/L calcium chloride aqueous solution was prepared using CaCl_2_ anhydrous powder (Merck‐Millipore). GG solution (600 µL) was added dropwise (1 drop/s) into 8 mL of CaCl_2_ solution in a 6‐well plate with shaking by hand. Then Ca‐GG hydrogel beads incorporate different concentrations of IL‐4 were obtained (Figure 1A). The beads were washed using PBS phosphate buffer saline (PBS, HyClone) thoroughly to remove excessive CaCl_2_. And then the beads were used in both in vitro and in vivo studies. The structure of Ca‐GG hydrogel was investigated by scanning electron microscope (SEM). And then Ca‐GG hydrogel beads with different IL‐4 concentrations were soaked in 2 mL DMEM with 10% FBS. After incubation at 37°C, the supernatant was collected at 1, 3, 5 and 7 days and sterilized using 0.2‐μm filter membranes for further use. To detect IL‐4 release, 1 mL supernatant was collected at the designated time points (1, 3, 5, 12, 24, 36, 72, 120, 168 hours), and 1 mL fresh medium was added to the sample. The IL‐4 concentration of all the samples was analysed by enzyme‐linked immunosorbent assay (ELISA, Bioswap).

### 2.2 Cell culture

Mouse BMSCs and the murine‐derived macrophage cell line RAW 264.7 (RAW) were used in this study. The BMSCs were isolated and cultured according to previous studies.^29^, ^30^ The RAW cells were cultured in DMEM (HyClone) supplemented with 10% FBS, 100 units/mL penicillin and 100 mg/mL streptomycin in a cell incubator.

### 2.3 Proliferation capacity assay

The proliferation capacity of RAW 264.7 cells and BMSCs was detected using a Cell Counting Kit‐8 (CCK‐8, Dojindo). We seeded 2000 RAW 264.7 cells in a 96‐well tissue culture plate. After 24 hours of incubation, we replaced the culture medium with 200 μL of conditioned media. Regular DMEM with 10% FBS was used as a control. At the designed time points (days 1, 2 and 3), the proliferation capacity of each well was tested using a CCK‐8 assay according to the manufacturer's protocol. Briefly, 100 μL DMEM including 10 μL CCK‐8 solution was added to each well at each time point and then incubated at 37°C for 1 hour. Then, the absorbance was measured at 450 nm.

The same assay was used to examine the proliferation capacity of BMSCs.

### 2.4 Cell viability assay

Cell viability within the Ca‐GG hydrogels and Ca‐GG + IL‐4 hydrogels was assessed using a Live & Dead Viability Assay Kit (KeyGEN Biotech). For the 3D culture of BMSCs, 4 × 10^4^ BMSCs were seeded in 1 mL Ca‐GG hydrogel and Ca‐GG + IL‐4 hydrogel in a 12‐well plate. Then, the samples were incubated in 2 mL fresh α‐MEM with 10% FBS. After 3 days of incubation, Live & Dead Viability staining was performed according to the protocol.

### 2.5 Macrophages polarization assay

Macrophages were seeded in 24‐well plates at a density of 1 × 10^3^ per well. The macrophages were firstly treated with 100 ng/mL lipopolysaccharide (LPS, Sigma‐Aldrich) and 10 ng/mL IFN‐γ (Peprotech) for 24 hours. And then the medium was replaced by regular medium (PC), Ca‐GG hydrogel bead‐conditioned medium (0 ng/mL), Ca‐GG + IL‐4 (100 ng/mL) hydrogel bead‐conditioned medium (100 ng/mL), Ca‐GG + IL‐4 (200 ng/mL) hydrogel bead‐conditioned medium (200 ng/mL) and Ca‐GG + IL‐4 (300 ng/mL) hydrogel bead‐conditioned medium (300 ng/mL). After 24 hours of cultivation, the identification of M1 and M2 macrophages was performed using immunofluorescence staining, ELISA, flow cytometry and qPCR.

### 2.6 Co‐culture of BMSCs and macrophages

To determine the effects of macrophages treated with different conditioned media on the osteogenic differentiation of BMSCs, a transwell co‐culture system was used. Macrophages were seeded on Hanging Cell Culture Inserts containing 0.4‐μm pores in each well (Millipore) at a density of 5 × 10^4^ per well in 6‐well plates. The macrophages were firstly treated using 100 ng/mL LPS and 10 ng/mL IFN‐γ for 24 hours and then incubated with different conditioned media (Figure 3A). BMSCs were seeded in flat‐bottom 24‐well transwell plates at a density of 5 × 10^3^ cells per well.

#### 2.6.1 BMSC apoptosis assay

Bone mesenchymal stem cell apoptosis was detected using an apoptosis detection kit (KeyGEN) according to the manufacturer's protocol. Briefly, BMSCs were harvested after 1 and 3 days of co‐culture with macrophages. Then, the BMSCs were resuspended using 1× annexin‐binding buffer (100 μL). After 30 minutes of incubation with 1 μL propidium iodide (PI, 100 μg/mL) and 5 μL Annexin V in the dark, the apoptosis rate of the BMSCs was analysed using flow cytometry. In addition, the total protein was harvested on days 1 and 3, and cleaved caspase 3 protein expression levels were detected using Western blotting.

#### 2.6.2 Osteogenic differentiation assay

Bone mesenchymal stem cells and macrophages were seeded in a Transwell co‐culture system. After 24 hours of incubation, the BMSC culture media were replaced with osteogenic medium (10 mmol/L β‐glycerophosphate, 50 μmol/L l‐ascorbic acid 2‐phosphate and 100 nmol/L dexamethasone). The alkaline phosphatase (ALP) activity was detected at day 7. After 2 weeks of induction, calcium deposition was assessed using Alizarin Red S (ARS) staining. The total RNA and protein were harvested after 7 days of co‐culture to determine the expression of osteogenesis‐related genes and proteins using qPCR and Western blot.

### 2.7 Activation of TGF‐β1/Smad signalling pathway

The total protein was harvested from the BMSCs after 5 days of co‐culture. To investigate the activation of the TGF/Smad signalling pathway in the BMSCs, the related proteins, including TGF‐β1 receptor (TGF‐β1R), phosphorylated Smad2(p‐Smad2) and phosphorylated Smad3(p‐Smad3), were analysed using Western blot. To further confirm the participation of the TGF/Smad pathway, we inhibited TGF‐β1R expression of BMSCs by siRNA. Two siRNA sequences were used in this study and the sequences were: 5′GGACCAUCCAUCCACUGAATT3′, 5′UUCAGUGGAUGGAU‐GGUCCTT3′ and 5′GCUCUGGUACUCUGGGAAATT3′, 5′UUUCCCAGAGUACCAGAGCT‐T3′. The TGF‐β1/Smad signalling pathway‐related proteins, including the TGF‐β1 receptor, p‐Smad2 and p‐Smad3, were analysed using Western blot.

### 2.8 In vivo animal experiment and histological evaluation

All animal care and experiments were conducted under the supervision of the Animal Research Committee of the West China School of Stomatology, Sichuan University (WCHSIRB‐D‐2016‐150). 12‐weeks‐old male SD rats were randomly divided into 3 groups: the negative control (NC) group, Ca‐GG hydrogel bead group and Ca‐GG + IL‐4 hydrogel bead group. The buccal mandible of the first molar was captured by tip forceps and extracted the molar to form a mandible bone defect. Then, either Ca‐GG hydrogel beads or Ca‐GG + IL‐4 (10 ng) hydrogel beads were implanted.^27^ We implanted 3 Ca‐GG hydrogel beads into the defects of the rats in Ca‐GG hydrogel bead group. And 3 Ca‐GG hydrogel beads loading 10 ng of IL‐4 were implanted into the defects of the rats in Ca‐GG + IL‐4 group. The mandibles were harvested 3 and 7 days after implantation for subsequent histopathology and immunohistochemistry of inducible nitric oxide synthase (iNOS, Abcam), CD206 (Abcam) and TNF‐α (Abcam). To detect cell apoptosis in surrounding tissue, a terminal deoxynucleotidyl transferase 2′‐deoxyuridine 5′‐triphosphate nick end labelling (TUNEL) assay was performed according to protocol.

### 2.9 Evaluation of osteogenesis in vivo

To evaluate the bone regeneration capacity of Ca‐GG + IL‐4 hydrogel bead in vivo, a critical‐sized bone defect was created in the rat mandible using a 5‐mm diameter trephine burr.^31^ Either Ca‐GG hydrogel beads or Ca‐GG + IL‐4 (10 ng) hydrogel beads were placed in the defects. We implanted 3 Ca‐GG hydrogel beads into the defects of the rats in Ca‐GG hydrogel bead group. And 3 Ca‐GG hydrogel beads loading 10 ng of IL‐4 were implanted into the defects of the rats in Ca‐GG + IL‐4 group. Nothing was placed in the defects of the control group. The mandibles were harvested 12 weeks after implantation. Micro‐CT measurement and immunohistochemistry for osteocalcin (OCN, Abcam) and Runx2 (ab23981, Abcam) were performed.

### 2.10 Statistical analysis

All data are expressed as mean values ± SD. We used GraphPad Prism 8.0 to compute statistical significance between 2 groups with 2‐tailed Student's *t* test. And the statistical significance between more than 2 groups was compared by one‐way ANOVA followed by Dunnett's multiple comparisons test. *P* < .05 was considered statistically significant.

## 3 RESULTS

### 3.1 Structure characteristic, cell viability, cell proliferation and IL‐4 release profile

SEM analysis showed that the micro‐structure of Ca‐GG was porous (Figure 1B), which facilitates the loaded IL‐4 to enter the surrounding tissues in vivo. The diameter of Ca‐GG hydrogel bead is (2.03 ± 0.11) mm (Figure 1C), and the porosity of Ca‐GG hydrogel is 74.14 ± 2.27%. The cumulative release profiles are plotted in Figure 1D. The conditioned media with or without IL‐4 did not significantly change the proliferation rates compared to the control media (Figure 1E). Live and dead staining results demonstrated that Ca‐GG hydrogel with or without IL‐4 had no significant effect on the ratio of live cells in the gels (Figure 1F).

### 3.2 Effect of conditioned media on macrophage polarization in vitro

**Figure 2:**
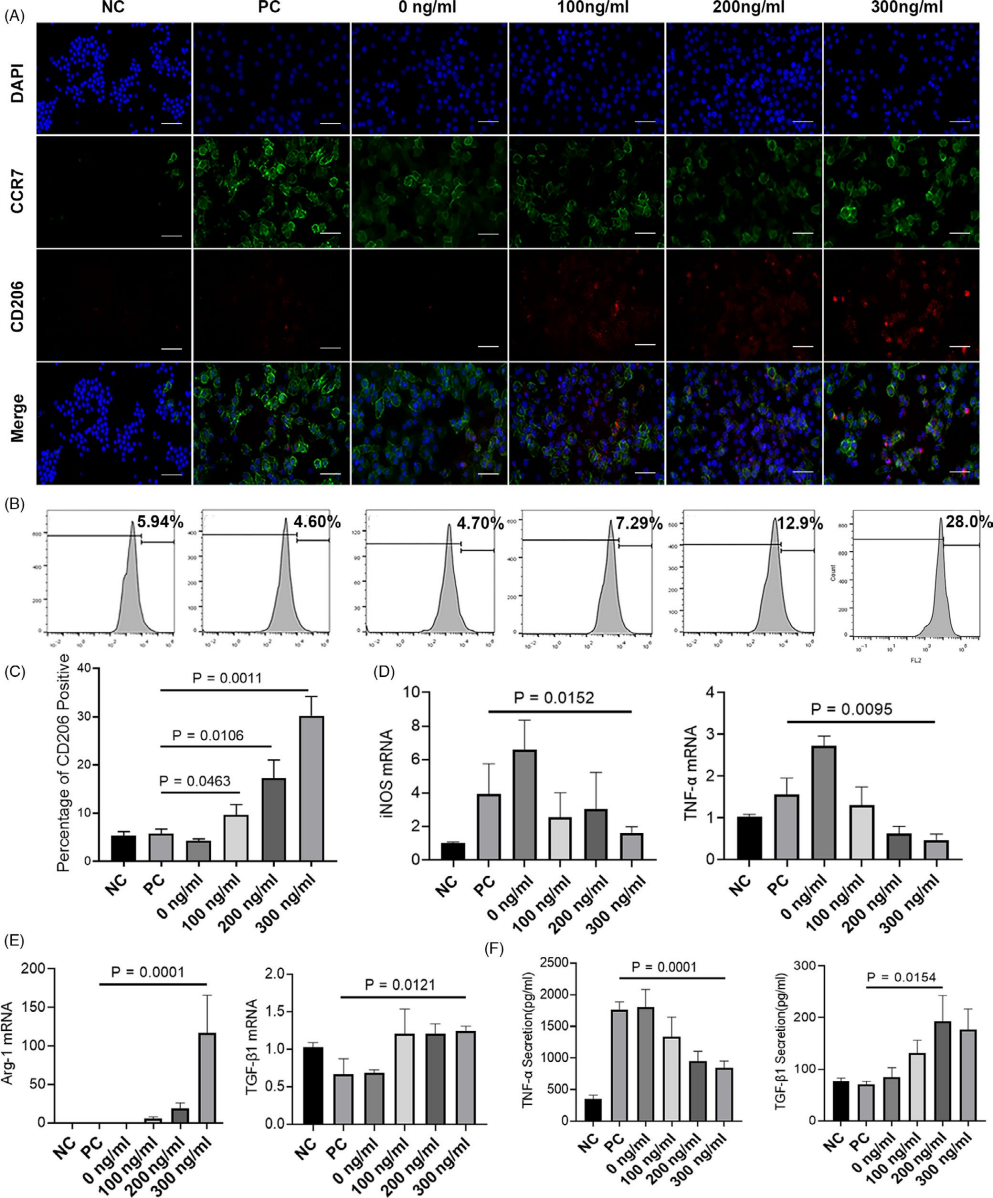
Ca‐GG + IL‐4 hydrogel bead‐conditioned media promote M2 Polarization of macrophages. A, M1‐related proteins (CCR7, green fluorescence) and M2‐related proteins (CD206, red fluorescence) in macrophages (immunofluorescence staining) following a 24 h cultured in different conditioned media (scale bar = 50 μm). B, Percentage of CD206 positive macrophages detected by Flow cytometry. C, Quantification of CD206 positive macrophages percentage (n = 3). D, Relative gene expression of M1 phenotype markers. E, Relative gene expression of M2 phenotype markers. F, Secretion levels of TNF‐α and TGF‐β1. NC, negative control; PC, regular medium; 0 ng/mL, Ca‐GG conditioned media; 100 ng/mL, Ca‐GG + IL‐4 (100 ng/mL) conditioned medium; 200 ng/mL, Ca‐GG + IL‐4 (200 ng/mL) conditioned medium; 300 ng/mL, Ca‐GG + IL‐4 (300 ng/mL) conditioned medium

Immunofluorescence staining results demonstrated that LPS + IFN‐γ treatment resulted in higher expression of CCR7, and macrophages cultured in Ca‐GG + IL‐4 hydrogel bead‐conditioned media were more positively stained for CD206 (Figure 2A). Flow cytometry analysis revealed that Ca‐GG + IL‐4 hydrogel bead‐conditioned media treatment increased the population of the M2 polarized macrophages. The percentage of M2 phenotype macrophages after cultured in Ca‐GG + IL‐4 (300 ng/mL)‐conditioned media reached 30.21 ± 3.22%, while in LPS + IFN‐γ alone group was barely detected (Figure 2B,C). qPCR analysis showed that high mRNA levels of M1‐related markers, including TNF‐α and iNOS, were generally induced by LPS and IFN‐γ (Figure 2D), whereas high levels of M2‐related markers (Arg‐1) were induced by the beads with IL‐4 (Figure 2E). The ELISA analyses also revealed that the Ca‐GG + IL‐4 hydrogel bead‐conditioned media resulted in a significant reduction in TNF‐α. The TNF‐α secretion in 300 ng/mL group was reduced to 838.85 ± 102.35 pg/mL, while the LPS + IFN‐γ alone group elicited 1762.8 ± 115.5271397 pg/mL. In contrast, the secretion of TGF‐β1 in the Ca‐GG + IL‐4 hydrogel bead groups was significantly higher than that of LPS + IFN‐γ alone group (Figure 2F).

**Figure 3:**
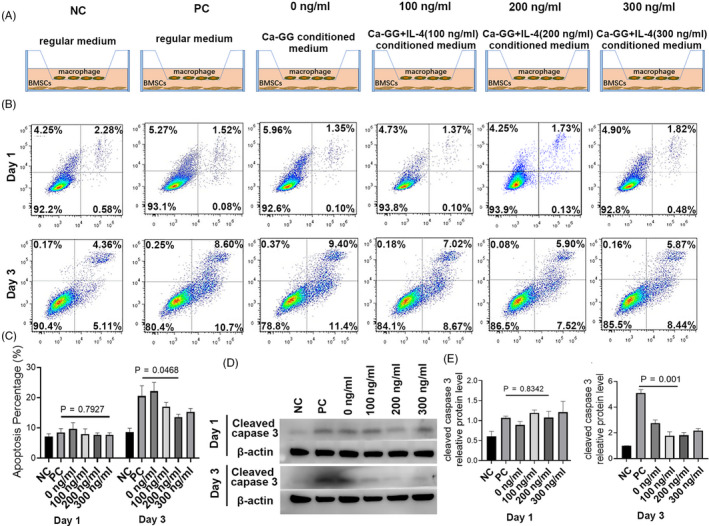
Ca‐GG + IL‐4 hydrogel bead‐conditioned medium reduced BMSCs apoptosis in co‐culture system. A, Schematic diagram of co‐culture system. NC, macrophages cultured with regular medium; PC, macrophages cultured with regular medium after 24 h of treated by LPS and IFN‐γ; other three groups were macrophages cultured with different conditioned medium after 24 h of treated by LPS and IFN‐γ. B, BMSCs apoptosis detected with Annexin V‐FITC/propidium iodide at day 1 and day 3 in co‐culture system. C, Quantification of BMSCs apoptosis rate (n = 3). D, Western blot analysis of cleaved Caspase 3 activity. E, Quantification analysis of cleaved Caspase 3 expression (n = 3)

### 3.3 Apoptosis of BMSC in co‐culture system

Annexin V‐FITC/propidium iodide (PI) double staining showed that no significant difference was observed among all the groups after 1 day of co‐culture (*P* > .05). The apoptotic rate of BMSCs in the Ca‐GG + IL‐4 (300 ng/mL) group (13.53 ± 0.78%) was significantly reduced compared with that of LPS + IFN‐γ alone group (20.53 ± 2.77%) after 3 day of co‐culture (Figure 3B,C). Cleaved caspase‐3 activation level of Ca‐GG + IL‐4 group was significantly reduced compared with that of LPS + IFN‐γ alone group after 3 days of co‐culture (Figure 3D,E).

### 3.4 Osteogenic differentiation of BMSCs in co‐culture system

**Figure 4:**
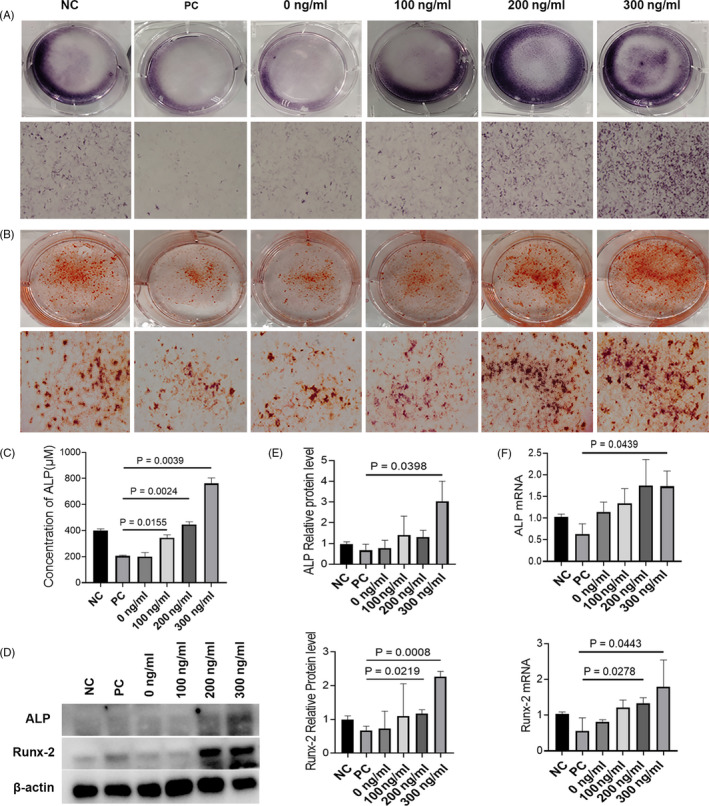
Ca‐GG + IL‐4 hydrogel bead‐conditioned media treated macrophages promote the osteogenesis of BMSCs. A, ALP staining of BMSCs after 7 d of co‐culture with different conditioned media treated macrophages. B, ARS staining of BMSCs after 21 d of co‐culture with different conditioned media treated macrophages. C, Quantitative analysis of ALP activity. D, Western blot analysis of ALP and Runx‐2 protein expression in BMSCs after 7 d of co‐culture with different conditioned media treated macrophages. E, Quantification analysis of the expression of ALP and Runx‐2 (n = 3). F, Real‐time qPCR analyses of ALP, Runx‐2 mRNA expression in BMSCs at day 7

ALP and ARS staining indicated that macrophages treated with Ca‐GG + IL‐4 hydrogel bead‐conditioned medium were beneficial for BMSC osteogenesis, whereas macrophages treated with LPS + IFN‐γ clearly inhibited osteogenic differentiation (Figure 4A‐C). The transcription levels of osteogenesis‐related genes were prominently increased by the activation of macrophages treated with Ca‐GG + IL‐4 hydrogel bead‐conditioned media, whereas macrophages treated with LPS + IFN‐γ elicited the opposite results (Figure 4D,E). The qPCR data were consistent with the following Western blotting results (Figure 4F).

### 3.5 The activation of TGF‐β1/Smad pathway in BMSCs

**Figure 5:**
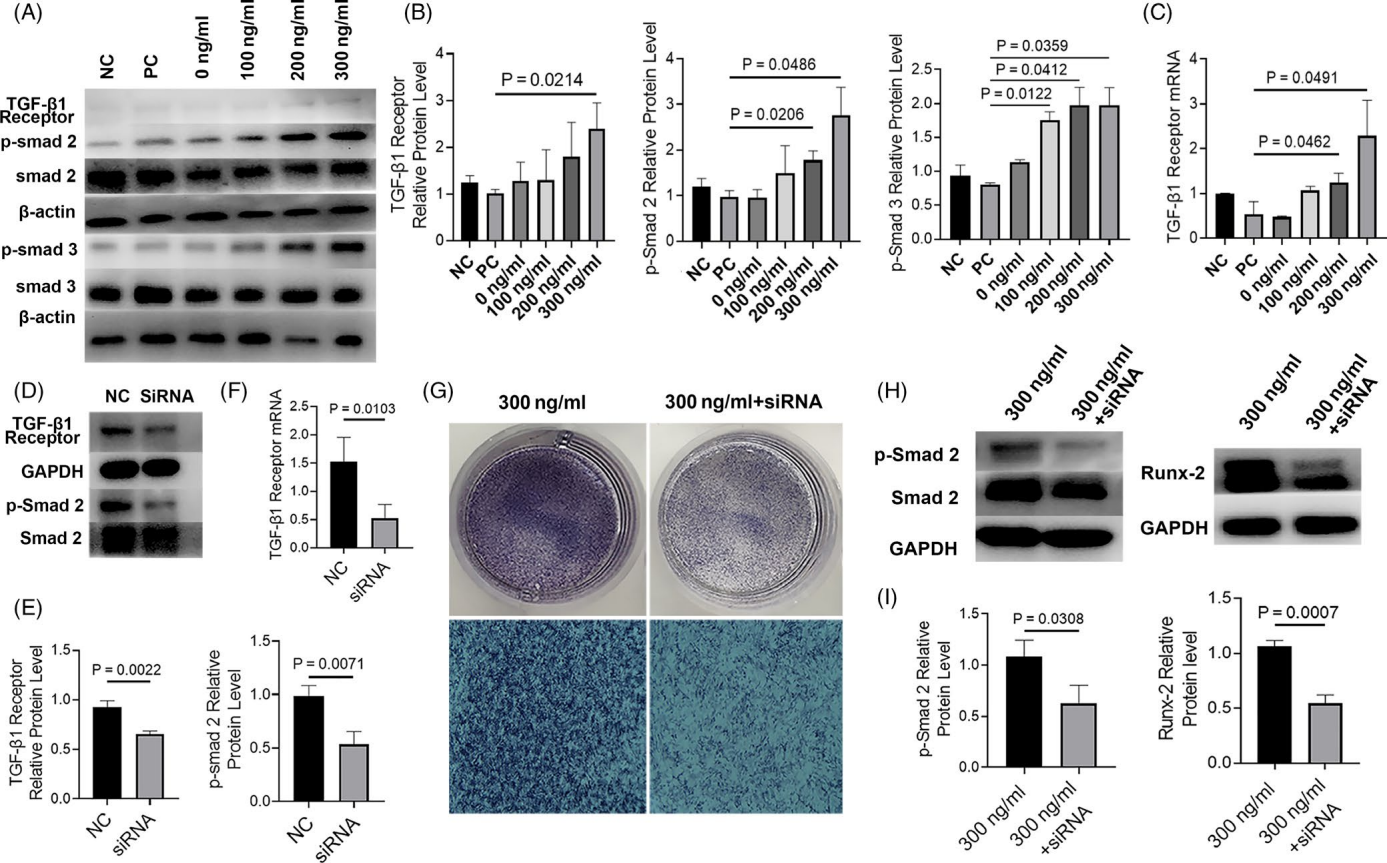
TGF‐β1/Smad signalling pathway was activated in BMSCs when co‐cultured with Ca‐GG + IL‐4 hydrogel bead‐conditioned media treated macrophages. A, Western blot analysis of TGF‐β1R, p‐Smad2 and p‐Smad3 expression in BMSCs at day 7. B, Quantification analysis of the expression of TGF‐β1R, p‐Smad2 and p‐Smad3 (n = 3). C, Real‐time qPCR analyses of TGF‐β1R expression of BMSCs at day 7. D, Western blot analysis of TGF‐β1R and p‐Smad2 in BMSCs after siRNA‐mediated inhibition of TGF‐β1R. E, Quantification analysis of the expression of TGF‐β1R and p‐Smad2. F, Real‐time qPCR analyses of TGF‐β1R expression of BMSCs siRNA‐mediated inhibition of TGF‐β1R. G, ALP staining of siRNA‐treated BMSCs after 7 d of co‐culture with Ca‐GG + IL‐4 (300 ng/mL) hydrogel bead‐conditioned media treated macrophages. H, Western blot analysis of p‐Smad2 and Runx‐2 of siRNA‐treated BMSCs after 7 d of co‐culture with Ca‐GG + IL‐4 (300 ng/mL) hydrogel bead‐conditioned media treated macrophages. I, Quantification analysis of the expression of p‐Smad2 and Runx‐2 in siRNA‐treated BMSCs (n = 3)

The protein expression of TGF‐β1R, p‐Smad2 and p‐Smad3 in Ca‐GG + IL‐4 groups was significantly increased compared with that of LPS + IFN‐γ alone group after 5 days of co‐culture (Figure 5A,B). And the expression levels of TGF‐β1R mRNA were similar to protein expression (Figure 5C). To further confirm the participation of the TGF/Smad pathway, we inhibited TGF‐β1R expression by siRNA and the knockout rate was 73.86 ± 7.01% (Figure 5D‐F). ALP staining showed that inhibition of the TGF/Smad pathway reduced the osteogenic capacity of BMSCs compared to that observed in the Ca‐GG + IL‐4 group (Figure 5G). The increased expression of osteogenic‐related genes in BMSCs induced by Ca‐GG + IL‐4 hydrogel bead‐conditioned media was reversed by the inhibition of the TGF‐β1/Smad pathway (Figure 5H,I).

### 3.6 Early‐stage host response and macrophage polarization in vivo

**Figure 6:**
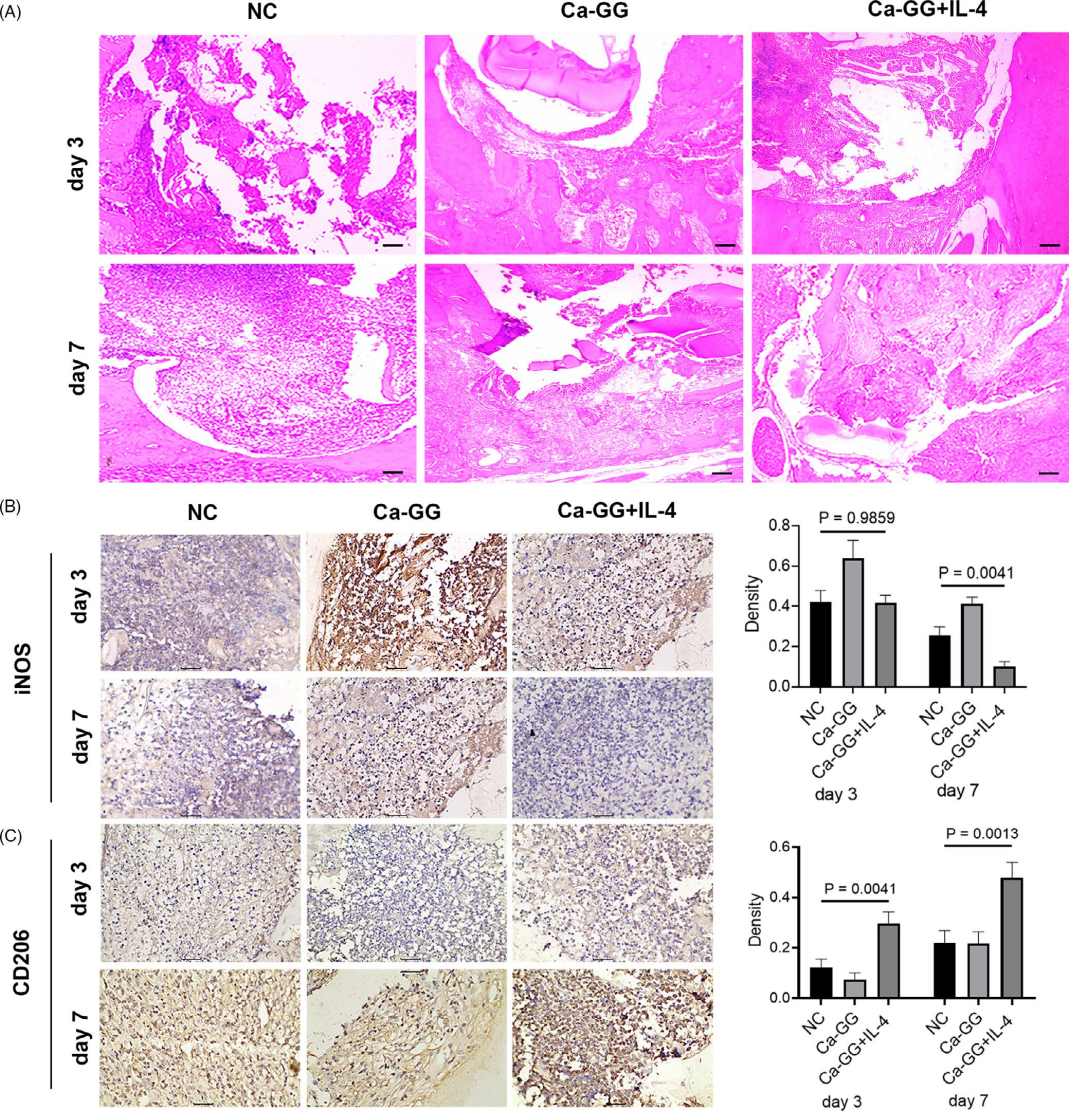
Ca‐GG + IL‐4 hydrogel beads promote M2 polarization of macrophages in vivo. A, H&E staining assessment of the tissue surrounding the Ca‐GG hydrogel beads 3 and 7 d post‐implantation. Scale bar = 1000 μm. *Undegraded Ca‐GG hydrogel beads. B, Immunohistochemical analysis of iNOS in the surrounding tissue 3 and 7 d post‐implantation. Scale bar = 100 μm. C, Immunohistochemical analysis of CD206 in the surrounding tissue 3 and 7 d post‐implantation. Scale bar = 100 μm

The tissue surrounding the implanted material presented significant inflammatory cell infiltration, which is a transient inflammatory response (Figure 6A). The analysis of iNOS‐positive macrophages revealed an overall reduction from day 3 to day 7 post‐implantation. A significant reduction in iNOS‐positive macrophages was observed in the Ca‐GG + IL‐4 group compared with the other 2 groups at day 7 post‐implantation, even though no significant difference was observed at day 3 post‐implantation (Figure 6B). The analysis of CD206‐positive macrophages showed that the Ca‐GG + IL‐4 hydrogel beads effectively polarized the macrophages towards the M2 phenotype at both day 3 and day 7 post‐implantation in vivo (Figure 6C).

### 3.7 Cell apoptosis assay in vivo

**Figure 7:**
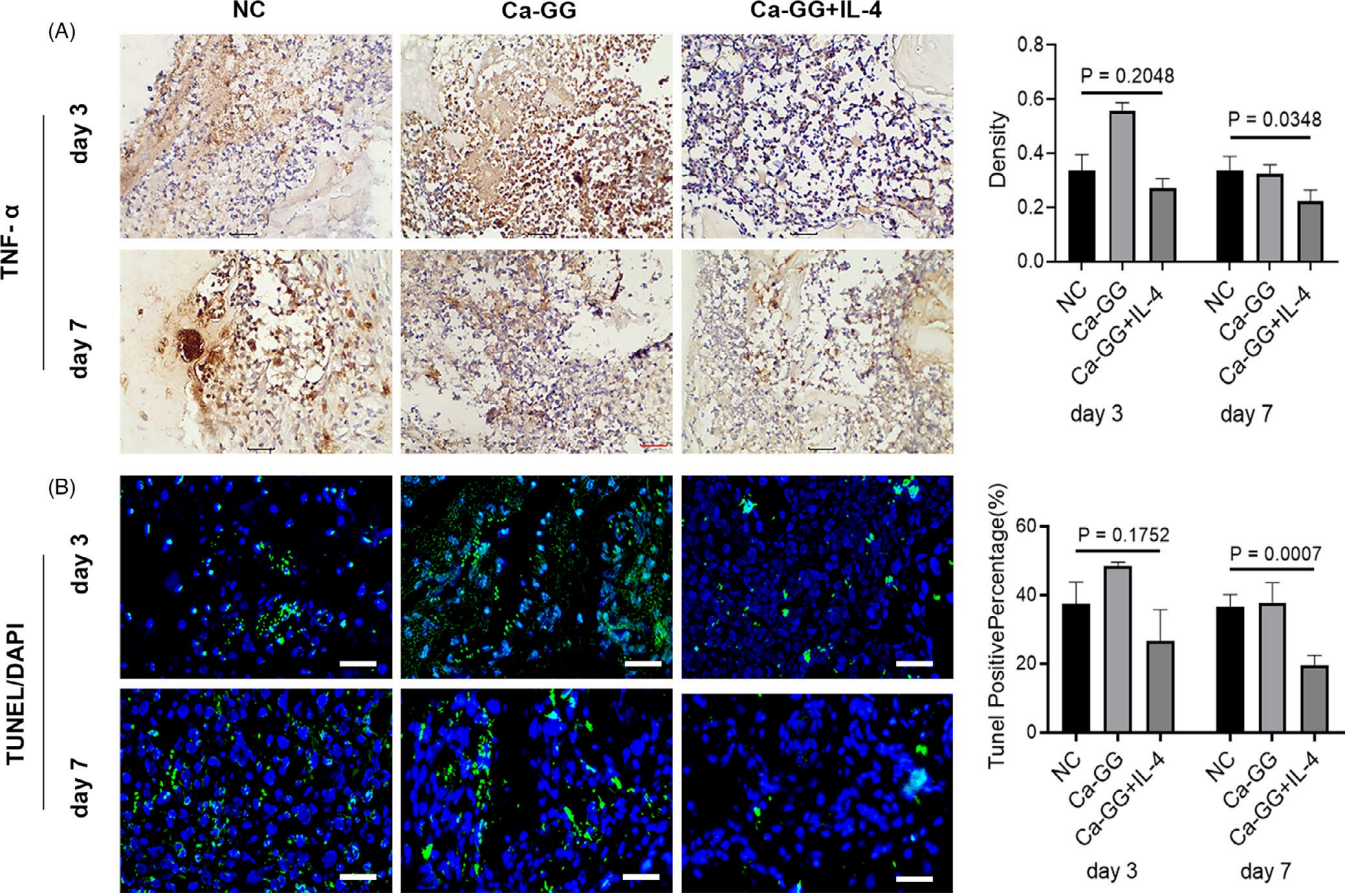
Immunomodulation via Ca‐GG + IL‐4 hydrogel beads reduced local tissue cell apoptosis at the early post‐implantation stage. A, Immunohistochemical analysis of the secretion of TNF‐α in the tissue surrounding the Ca‐GG hydrogel beads at 3 and 7 d post‐implantation. B, TUNEL examination of cell apoptosis in the surrounding tissue. Green fluorescence represents apoptotic cells. Scale bar = 100 μm

Immunohistochemical analysis showed that a lower level of TNF‐α expression was observed in Ca‐GG + IL‐4 group at day 7 post‐implantation compared to that of other 2 groups (Figure 7A). However, no significant difference was observed in the expression of TNF‐α at day 3 post‐implantation. TUNEL assay results demonstrated that a significantly low level of cell apoptosis was observed at 7 days after implantation in Ca‐GG + IL‐4 group. Similarly, no significant difference was observed at day 3 post‐implantation (Figure 7B).

### 3.8 Bone regeneration capacity analysis

**Figure 8:**
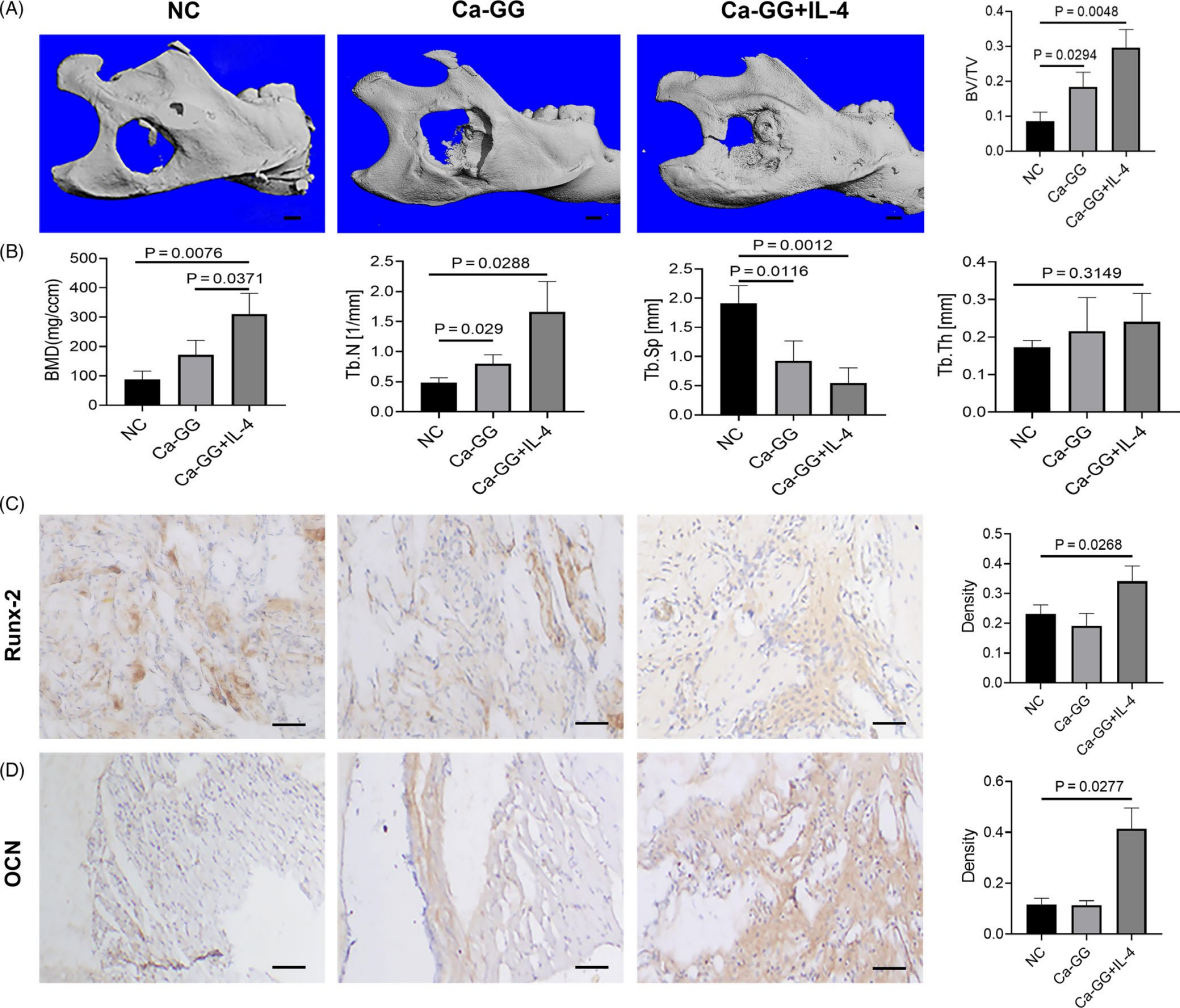
Ca‐GG + IL‐4 hydrogel beads promote in mandible defects regeneration in vivo. A, Micro‐CT 3D reconstruction images showed the best bone defect healing outcome in Ca‐GG + IL‐4 hydrogel bead group. Scale bar = 5 mm. B, Quantification analysis of bone mineral density, trabecular number, trabecular thickness and trabecular separation. C, Immunohistochemical analysis of Runx‐2 in bone defect area 12 wk after implantation. Scale bar = 50 μm. D, Immunohistochemical analysis of OCN in bone defect area 12 wk after implantation. Scale bar = 50 μm

Micro‐CT examination showed that newly formed bone in Ca‐GG + IL‐4 group expanded to most of the bone defect area. The ratio of new BV to total BV (BV/TV, %) was 8.56 ± 2.16% in NC group, 18.44 ± 3.40% in GG group, and 29.63 ± 4.32% in Ca‐GG + IL‐4 group. The quantification analysis of the newly formed bone showed that the Ca‐GG + IL‐4 group had up to 3.46‐fold more newly formed bone volume than that of NC group (Figure 8A). And the Quantification analysis of bone mineral density, trabecular number and trabecular separation showed that Ca‐GG + IL‐4 hydrogel beads achieve superior bone defect repair efficacy (Figure 8B). Immunohistochemical analysis of Runx‐2 and OCN showed that the osteogenetic activity of the Ca‐GG + IL‐4 group was higher than that of other 2 groups (Figure 8C,D).

## 4 DISCUSSION

Host immune reaction to tissue engineering scaffold has been recognized as a crucial factor that determines therapeutic efficacy.^32^, ^33^ This study presented evidence that immunomodulation patterns play a crucial role in osteogenesis during bone defect regeneration. The effects of the Ca‐GG + IL‐4 hydrogel beads on macrophage polarization were investigated both in vitro and in vivo. The immunofluorescence staining and qPCR results revealed that Ca‐GG + IL‐4 hydrogel beads could attenuate the M1 polarization of macrophages and activate M2 polarization, which improved the osteogenesis of BMSCs in the co‐culture system in vitro. Moreover, the apoptosis of BMSCs in the co‐culture system induced by M1 macrophage‐secreted TNF‐α was significantly reduced in the Ca‐GG + IL‐4 hydrogel bead group compared with other groups. The loaded IL‐4 could promote M2 macrophage polarization and increase TGF‐β1 expression level, which activates the TGF‐β1/Smad signalling pathway in BMSCs and promotes osteogenic differentiation. Moreover, the immunohistochemical analysis of iNOS‐positive macrophages revealed an overall reduction from day 3 to day 7 post‐implantation in vivo. The quantitative analysis of iNOS IHC staining showed that the implantation of Ca‐GG hydrogel beads leads to the M1 polarization of macrophages. However, the IL‐4 incorporated in the Ca‐GG hydrogel beads could ameliorate the excess M1 polarization caused by the Ca‐GG hydrogel beads. The immunohistochemistry analysis of CD206‐positive macrophages showed that the IL‐4 incorporated in the Ca‐GG hydrogel beads effectively polarized the macrophages towards the M2 phenotype at both day 3 and day 7 post‐implantation in vivo. And a significantly low level of cell apoptosis was observed at 7 days after implantation in Ca‐GG + IL‐4 group. Furthermore, Micro‐CT analysis of the newly formed bone showed that the Ca‐GG + IL‐4 group had up to 3.46‐fold more newly formed bone volume than the NC group at 12 weeks post‐surgery which indicated that Ca‐GG + IL‐4 hydrogel beads significantly improved bone defect regeneration in vivo. The results of the immunohistochemical analysis of OCN and Runx2 were consistent with the micro‐CT results. Collectively, this study demonstrated that the coordinated crosstalk between macrophages and BMSCs and the proper regulation of macrophage polarization at the early injury stage play a key role in bone regeneration process.

Bone regeneration involves a series of complex and continuous physiological processes.^34^ Several kinds of cells, including macrophages, stem cells, osteoblasts, osteoclasts and endothelial progenitor cells, are recruited from their local niches and then develop into mature bone tissues and vessels.^35^, ^36^ The reaction between tissue engineering scaffold and surrounding cells may determine the therapeutic efficacy; therefore, the scaffold material always delivers a drug to manage the host response, such as immunomodulatory drugs.^37^, ^38^ M1 phenotype macrophages secrete factors such as SDF‐1, which is involved in BMSC migration, while M2 phenotype macrophages produce factors such as TGF‐β1, BMP‐2 and VEGF, which promote bone regeneration.^39^ Increasing studies have shown that the precise and sequential polarization of M1 and M2 macrophages accelerates the tissue regeneration process.^40^, ^41^ Here, the data in this study showed that the M1 phenotype rapidly infiltrated into the bone defect area at the early stage of injury, while the M2 phenotype showed a gradually increasing trend. Hydrogel is an ideal material that can effectively mimic the ECM microenvironment and consequently promote cell migration and regeneration outcomes.^42^ Some studies have shown that Ca‐GG hydrogels are functional bone‐bioactive materials that are not only able to mineralize but also compatible with efficient drug delivery applications in a wide range of molecular weights.^18^, ^43^ Previous study showed that proper delivery of IL‐4 can generate the most preferable M1/M2 macrophage profile, resulting in a pro‐healing microenvironment coupled with enhanced downstream osteogenesis.^27^ Therefore, we developed a strategy that using Ca‐GG hydrogel bead to delivers IL‐4 for repair of jaw defect.

The therapeutic outcome of mandibular defects in this study showed that the sustained local release of IL‐4 from the bone‐bioactive Ca‐GG hydrogel beads is effective for bone regeneration in vivo. The success of in situ bone regeneration is based on the coordinated reaction between macrophages and BMSCs. Precise and sequential M2 polarization of macrophages at early stage promotes the osteogenic differentiation of BMSCs and then accelerates bone regeneration in the defect area.^27^, ^40^ Generating a preferable M1/M2 profile led to a pro‐healing microenvironment and superior bone regeneration efficacy. However, macrophages and BMSCs cannot represent the complex cellular microenvironment in vivo. Multiple cells, including B cells and T cells, also synergistically participate in the bone regeneration process. Therefore, more studies regarding these cells should also be carried out. Furthermore, based on the effect of this bone‐bioactive hydrogel composite in rats, the therapeutic efficacy needs to be verified in further research on larger animals, such as pigs and dogs. The bone architecture of larger animals is more similar to that of humans, and the therapeutic efficacy in these models would be more convincing.

## 5 CONCLUSION

In conclusion, this study demonstrated the therapeutic efficacy of Ca‐GG + IL‐4 hydrogel beads on bone regeneration. The study demonstrated that the IL‐4 incorporated in the Ca‐GG hydrogel beads promotes M2 polarization of macrophages and then improves the osteogenic differentiation capacity of BMSCs by triggering the TGF‐β1/Smad signalling pathway in vitro. The implantation of Ca‐GG hydrogel beads with proper doses of IL‐4 could result in the precise polarization of M1 and M2 macrophages, leading to a microenvironment that promotes tissue regeneration in vivo. These results showed that the combined effect of bone‐bioactive scaffold and immunomodulatory agents potentiates the ultimate therapeutic efficacy of bone regeneration and reduces the side effects and resistance to drugs. In addition, the results provide evidence for the further application of bone‐bioactive and immunomodulatory bone substitute material strategies in bone regeneration.

## CONFLICT OF INTERESTS

The authors have declared that no competing interest exists.

## AUTHOR CONTRIBUTIONS

Jian Pan and Wei Jing contributed substantially to the design of the experiments. Jiankang Zhang conducted all experiments and wrote the manuscript. Haitao Shi, Nian Zhang and Liru Hu assisted in conducting experiments and data analysis.

## Data Availability

The data used to support the findings of this study are available from the corresponding author upon request.
